# Botulinum Toxin for Chronic Migraine: Beyond Headache Reduction and Toward Possible Cognitive Benefits

**DOI:** 10.3390/toxins18040153

**Published:** 2026-03-24

**Authors:** Mauro Silvestrini, Giovanna Viticchi, Sergio Salvemini, Gioacchino De Vanna, Marco Bartolini, Simona Luzzi

**Affiliations:** Neurological Clinic, Marche Polytechnic University, Via Conca 71, 60020 Ancona, Italy; g.viticchi@univpm.it (G.V.); sergio.salvemini@ospedaliriuniti.marche.it (S.S.); gioacchino.devanna@ospedaliriuniti.marche.it (G.D.V.); m.bartolini@univpm.it (M.B.); s.luzzi@univpm.it (S.L.)

**Keywords:** botulinum toxin, chronic migraine, cognition, working memory, processing speed, central sensitization, cortical inhibition, neuropsychology

## Abstract

Chronic migraine (CM) is a debilitating neurological disorder characterized not only by persistent and severe pain, but also by substantial cognitive dysfunction that affects attention, working memory, processing speed, and executive functions. These neuropsychological disturbances are likely influenced by overall disease burden and are further modulated by affective comorbidities, sleep disturbances, and medication overuse. OnabotulinumtoxinA (BoNT-A) is an established preventive therapy for CM, supported by strong evidence of both efficacy and safety. This narrative review synthesizes findings from studies examining the relationship between BoNT-A treatment and domain-specific cognitive improvements in CM. It also outlines the potential pathophysiological mechanisms underlying these effects, summarizes the limitations of the existing literature, and highlights priorities for future research. Current evidence suggests that BoNT-A may confer neurocognitive benefits, particularly in working memory and processing speed, and that these improvements may occur partly independently of reductions in headache frequency. These favorable cognitive effects appear to be plausibly linked to decreased nociceptive “noise” and improved cortical inhibition, potentially mediated through modulation of central sensitization, nociceptive signaling, and affective states.

## 1. Introduction

Chronic migraine (CM) is a highly disabling primary headache disorder, defined as headache occurring on more than 15 days per month for over 3 months, with at least 8 days showing features of migraine with or without aura [1]. Recent reviews have further emphasized its global burden and therapeutic relevance, confirming CM as one of the leading causes of disability worldwide and reaffirming the central role of onabotulinumtoxinA (BoNT-A) as a validated preventive option [2]. Epidemiological data indicate that CM remains highly prevalent among working-age adults, contributing substantially to years lived with disability and highlighting the urgent need to address its associated cognitive impairments [3]. BoNT-A is an effective preventive therapy, consistently reducing monthly migraine days (MMDs) and demonstrating a favorable safety profile in both randomized trials and long-term real-world studies. According to a Cochrane systematic review, BoNT-A decreases migraine frequency by approximately two days per month compared with placebo, with a well-established tolerability profile [4]. Longitudinal observational cohorts further confirm sustained clinical improvement and low discontinuation rates [5]. Beyond the burden of pain, CM patients frequently report subjective cognitive symptoms, including processing-speed impairment, reduced working-memory efficiency, and challenges in executive functions, all of which significantly interfere with daily activities. These cognitive difficulties may persist even between attacks and can rank second only to pain as a major source of disability [6]. Evidence suggests that such deficits may improve with effective preventive treatment, including BoNT-A, although cognitive changes do not always correlate directly with headache reduction [7,8]. However, reported findings vary considerably depending on study methodology, assessment timing relative to the migraine cycle, and confounding factors such as anxiety, depression, sleep disturbance, and medication overuse [9]. The aim of this narrative review is to provide a critical synthesis of the available evidence, examining potential neurophysiological mechanisms, identifying current limitations in the literature, and outlining priorities for future research to better understand the effects of BoNT-A on cognitive functioning in CM.

## 2. Methods

We searched PubMed/MEDLINE, Scopus, and Web of Science from inception to 31 January 2026, with updates in June and October 2025, using MeSH terms and free-text keywords related to chronic migraine, onabotulinumtoxinA/botulinum toxin A, cognition/neuropsychology, central sensitization, cortical inhibition/excitability, and functional connectivity. Two reviewers independently screened titles, abstracts, and full-text articles (English; adult CM populations) and included clinical and mechanistic human studies of BoNT-A that reported cognitive outcomes or closely related interictal domains. Small case reports, pediatric samples, and non-CM populations were excluded. Extracted data included cognitive domains assessed, timing of evaluation relative to the injection cycle or migraine phase, affective covariates, and headache- or disability-related measures. This work is a narrative review and therefore does not follow the methodological rigor of a systematic review. Although a structured search strategy was applied, full reproducibility cannot be ensured, and some degree of selection bias may be present. Findings should thus be interpreted as a qualitative synthesis rather than a systematic evaluation.

## 3. Results

### 3.1. Cognitive Dysfunction in Chronic Migraine

Cognitive dysfunction is increasingly recognized as a core component of CM, extending its impact far beyond head pain. Patients commonly report difficulties in attention, processing speed, working memory, and executive functions, all of which can significantly impair daily activities and overall quality of life. These cognitive disturbances are typically multifactorial, influenced by the cumulative disease burden as well as psychiatric comorbidities, sleep disruption, and medication overuse. Real-world evidence indicates that individuals with CM experience poorer health-related quality of life and greater disability compared with those with episodic migraine, frequently presenting with cognitive symptoms such as poor memory and reduced cognitive efficiency [10]. Prospective studies also reveal measurable deficits in reaction time and working-memory accuracy, reinforcing the presence of objective neuropsychological impairment in CM [7]. Importantly, these deficits may persist even between headache episodes, suggesting an ongoing pathophysiological process affecting cortical networks [5,7,8]. Affective states play a substantial role as well: anxiety and depressive symptoms are known to contribute to cognitive inefficiency, and reductions in anxiety have been correlated with improvements in working-memory accuracy [4,8]. Broader investigations of interictal burden further highlight that cognitive slowing, fatigue, and photophobia are major contributors to disability and tend to improve with effective treatment [11]. Taken together, the available evidence supports the view that cognitive dysfunction in CM is a clinically meaningful and multidimensional phenomenon that warrants systematic assessment and targeted therapeutic strategies.

### 3.2. Evidence for Cognitive Benefits of OnabotulinumtoxinA

Multiple studies have explored the potential of therapeutic interventions to mitigate cognitive deficits in patients with CM. Among available preventive options, BoNT-A has emerged not only as one of the most rigorously validated treatments for reducing headache frequency, but also as a promising candidate for improving cognitive performance through both direct and indirect mechanisms. Growing evidence suggests that the therapeutic role of BoNT-A extends beyond pain reduction and may contribute to the restoration of neurocognitive functioning in individuals affected by this highly disabling condition. A key contribution comes from the prospective real-world study by Ho et al., which provided some of the earliest objective evidence that BoNT-A can improve reaction time, processing speed, and working-memory accuracy within 6 to 12 weeks of treatment [8]. Notably, these improvements did not correlate with changes in headache frequency, indicating that the cognitive benefits may operate, at least in part, through mechanisms independent of nociceptive modulation. This finding aligns with the hypothesis that cognitive dysfunction in CM arises from widespread dysregulation of cortical processing and central sensitization. Additional support is provided by Sánchez-Huertas et al., who investigated the interictal burden of CM—a multidimensional cluster of symptoms including cognitive slowing, fatigue, photophobia, and mood disturbances—and found significant reductions in these impairments following BoNT-A therapy [11]. By relieving symptoms that interfere with cognitive efficiency, BoNT-A appears to facilitate functional recovery in cognitive domains that remain impaired even during headache-free periods. Recent real-world studies involving large patient cohorts (e.g., 579 individuals followed for up to 11 years) further reinforce the long-term safety and sustained clinical benefits of BoNT-A, showing consistent reductions in headache burden and disability with stable tolerability profiles [5]. Complementary analyses from 2024 also suggest that improvements in anxiety, depression, allodynia, and bruxism may contribute indirectly to enhanced cognitive efficiency by modulating affective and sensory pathways, underscoring the multidimensional impact of BoNT-A [7]. Mechanistic studies published in 2024–2025 add further plausibility by describing potential interactions between peripheral CGRP-mediated pathways, satellite glial cell signaling, and trigeminal sensory processing, offering biological support for BoNT-A’s influence on nociceptive and attentional networks [12]. Long-term observations, such as those from Santoro et al., also demonstrate sustained reductions in monthly headache days, medication overuse, and disability for up to 11 years, with more than 60% of patients improving from severe to minimal disability levels [5]. Additional evidence pointing toward BoNT-A’s cognitive benefits comes from work showing that improvements in emotional regulation may mediate some of its neuropsychological effects. For instance, Ilgaz Aydinlar et al. found that BoNT-A significantly improved migraine-related disability, anxiety, depression, and sleep quality across diverse patient profiles, suggesting broader improvements in neurological well-being that may extend to cognitive domains [7]. Meta-analytic findings further support the superiority of BoNT-A over placebo in reducing chronic migraine attacks and migraine-related disability, indirectly reinforcing its potential to improve cognitive outcomes by lowering overall disease burden [13]. Although these studies were not primarily designed to measure cognitive endpoints, the consistent association between reduced disability and improved neuropsychological functioning is well documented in CM, supporting the plausibility of secondary cognitive benefits. Overall, the convergence of clinical, observational, and mechanistic evidence suggests that BoNT-A may meaningfully contribute to cognitive improvement in chronic migraine. However, the precise pathways remain insufficiently understood, and cognitive outcomes continue to be underrepresented in large-scale clinical trials. Future research should incorporate longitudinal neuropsychological assessments, functional neuroimaging, and biomarker-based approaches to clarify the magnitude, specificity, and durability of these cognitive effects. Nevertheless, current evidence strongly indicates that the therapeutic impact of BoNT-A extends beyond pain modulation, making cognition an increasingly relevant domain in the comprehensive management of CM. A summary of the results of the selected studies has been added in Table 1. It is important to emphasize, however, that current evidence regarding cognitive outcomes remains preliminary. Most findings originate from small observational cohorts or rely on indirect endpoints, making conclusions about direct cognitive enhancement tentative. Although some studies suggest that cognitive improvements may occur partly independently of headache reduction, this evidence remains limited. Therefore, the extent to which cognitive benefits are truly independent of analgesic effects is uncertain, and current findings should not be generalized beyond the specific contexts of the available studies.

### 3.3. Mechanisms Underlying Cognitive Gains in BoNT-A Botulinum Toxin–Treated Chronic Migraine

#### 3.3.1. BoNT-A Exerts Analgesic and Neuromodulatory Effects That Extend Beyond Its Classical Action on Motor Terminals, Primarily Through the Cleavage of SNAP-25

Even partial SNAP-25 cleavage can substantially attenuate presynaptic neurotransmitter release and dampen hyperexcitable sensory circuits, leading to reduced excitatory neurotransmission [14]. This inhibition also decreases the release of algogenic neuropeptides such as CGRP and substance P, both of which are key mediators of peripheral sensitization and nociceptive signaling [15]. By suppressing excessive excitatory input and neuropeptidergic activity, BoNT-A reduces sensory “noise” that would otherwise interfere with cognitive networks. This reduction may facilitate more efficient filtering of irrelevant stimuli and help stabilize cognitive functioning in individuals with chronic pain and sensory hyperexcitability [16]. Mechanistic work further strengthens the rationale for BoNT-A’s potential cognitive benefits. Pozo-Rosich et al. synthesized 25 years of mechanistic and clinical evidence, demonstrating that BoNT-A modulates both peripheral and central nociceptive pathways, reduces central sensitization, and restores cortical inhibitory tone [17]. This re-establishment of inhibitory balance may help prevent the aberrant cortical activation believed to contribute to cognitive impairment in CM. However, interpretation of these mechanistic links must be tempered by the limitations of available clinical evidence. Cochrane systematic analyses indicate that most randomized trials of BoNT-A for migraine are relatively short—typically no longer than nine months—restricting the ability to evaluate cognitive outcomes, which often require long-term follow-up to detect durable changes in processing speed, attention, and executive functions [4]. Neurophysiological studies using transcranial magnetic stimulation (TMS) provide additional insight, showing that patients with CM exhibit abnormal cortical excitability and impaired intracortical inhibition compared with both episodic migraine and healthy controls. Valente et al. reported that BoNT-A partially normalizes these aberrant patterns after repeated treatment cycles, supporting the hypothesis that BoNT-A promotes a more physiologically stable cortical environment conducive to cognitive improvement [18]. When considered alongside clinical data demonstrating improvements in interictal symptoms, affective states, and functional disability, these mechanistic observations collectively suggest that BoNT-A influences a broader neurobiological network than traditional analgesic therapies.

#### 3.3.2. Modulation of Functional Brain Connectivity

Functional MRI (fMRI) studies indicate that BoNT-A may modulate large-scale neural networks involved in attentional control and sensory processing, producing measurable changes in functional connectivity that parallel clinical improvements [19]. Although the precise mechanisms through which BoNT-A influences brain-network dynamics remain under investigation, evidence from the functional-connectivity literature shows that attentional and salience-related networks—such as the insula-based salience network and prefrontal regulatory circuits—are highly sensitive to alterations in peripheral and central sensory input [20,21]. Anxiety-related network signatures, particularly reduced connectivity within key salience-network nodes such as the supramarginal gyrus, insula, and anterior cingulate cortex, have been associated with affective dysregulation and increased sensory responsiveness [22]. These findings provide a neurobiological framework for understanding how the reduction in peripheral nociceptive drive and sensory “noise” by BoNT-A may secondarily normalize connectivity within circuits governing attention allocation, sensory filtering, and emotional regulation. Such network-level recalibration aligns with the clinically observed reductions in anxiety and improvements in overall symptom burden following BoNT-A treatment, supporting the hypothesis that its therapeutic effects extend beyond local neuromuscular actions to include central functional reorganization within higher-order cognitive and affective networks [23]. Nevertheless, these mechanistic interpretations should be approached cautiously. Although several neurobiological pathways plausibly link BoNT-A administration to enhanced cognitive efficiency, the causal chain remains hypothetical. Most mechanistic evidence is based on preclinical or indirect human findings, and no study to date has conclusively demonstrated that these pathways directly produce measurable cognitive improvements in patients with CM.

#### 3.3.3. Affective Modulation

Increasing evidence indicates that improvements in affective symptoms—particularly anxiety and depression—may mediate cognitive gains following BoNT-A treatment, independently of its effects on headache. Clinical studies across multiple movements and psychiatric disorders consistently show that BoNT-A reduces anxiety and depressive symptoms, and these mood improvements often occur even in the absence of substantial changes in motor severity, suggesting partially independent central or psychophysiological mechanisms [24]. Retrospective psychiatric data and systematic reviews similarly demonstrate that BoNT-A can alleviate depressive symptoms across several diagnostic categories and maintain therapeutic benefits over repeated treatment cycles, supporting its potential role as an adjunctive intervention for depression [25,26,27]. Additional evidence from studies in cervical dystonia indicates that BoNT administration not only reduces anxiety and improves quality-of-life measures but is also associated with enhancements in certain cognitive domains, further reinforcing the link between affective and cognitive pathways [28]. Large-scale post-marketing analyses, along with reviews and meta-analyses, also reveal robust antidepressant effects of BoNT across a variety of indications and injection sites, strengthening the view that BoNT-A influences affective processes in ways not solely attributable to symptom improvement in the underlying condition [29,30,31]. Consistent with these findings, Ho et al. reported that improvements in working-memory accuracy correlated more strongly with reductions in anxiety than with changes in headache activity, suggesting that BoNT-A–related cognitive enhancement may be partially driven by its ability to reduce affective symptoms known to impair attention, processing speed, and executive functioning [8].

### 3.4. Limitations of the Current Evidence

#### 3.4.1. Methodological Heterogeneity and Limited Cognitive Endpoints

Current research on the cognitive effects of onabotulinumtoxinA (BoNT-A) in chronic migraine (CM) is limited by substantial methodological heterogeneity. Most available studies were not primarily designed to assess cognitive outcomes, resulting in inconsistent use of neuropsychological instruments and limited incorporation of validated cognitive endpoints. For example, many investigations focus predominantly on headache-related outcomes and rely on indirect markers—such as disability scores, affective symptoms, or interictal burden—to infer cognitive change, which reduces the interpretability and specificity of the findings [6,7,9]. Cochrane systematic analyses further highlight that most randomized trials evaluating BoNT-A for migraine have relatively short follow-up periods (typically no longer than nine months), a constraint that critically limits the assessment of cognitive outcomes, which generally require long-term observation to detect meaningful changes [4]. Moreover, the few studies that include objective cognitive testing often suffer from small sample sizes and brief follow-up intervals, reducing statistical power and limiting the generalizability of results [8]. Collectively, this lack of standardized methodology makes it difficult to determine the magnitude, specificity, and durability of any potential cognitive benefits associated with BoNT-A.

#### 3.4.2. Confounding Clinical and Affective Variables

The interpretation of cognitive outcomes in CM is further complicated by the presence of multiple confounding factors. Anxiety, depression, sleep disturbances, and medication overuse—each highly prevalent in CM—exert independent negative effects on attention, executive functions, and working memory [9]. Evidence indicates that improvements in cognition following BoNT-A treatment may correlate more strongly with reductions in anxiety or affective burden than with changes in headache frequency, making it difficult to isolate potential direct neuromodulatory effects of the intervention [7,8]. Cognitive functioning in CM also fluctuates across ictal, peri-ictal, and interictal phases. However, most studies do not standardize the timing of cognitive assessments relative to the migraine cycle, introducing substantial intra-individual variability [6,7]. These uncontrolled confounders significantly limit causal inference. Taken together, they suggest that cognitive changes observed after BoNT-A treatment may reflect improvements in mood, sleep, or medication overuse rather than direct effects on cognitive networks.

#### 3.4.3. Limited Longitudinal and Mechanistic Data

Although long-term observational studies demonstrate persistent benefits of BoNT-A on headache frequency and disability, they rarely include cognitive endpoints, leaving long-term cognitive trajectories inadequately characterized [5]. Mechanistic investigations offer plausible biological explanations for potential cognitive improvements such as modulation of cortical excitability, restoration of inhibitory tone, and reduction in central sensitization—but these findings remain indirect and cannot establish causal relationships with cognitive outcomes [17,18]. The lack of multimodal study designs integrating neuropsychological assessment, functional neuroimaging, and biomarker analysis represents an important evidence gap and limits our understanding of how BoNT-A may influence higher-order cognitive processes.

#### 3.4.4. Inconsistent Experimental Designs and Variability Across Studies

Heterogeneity in study design further weakens the overall evidence base. Variations in treatment intervals, dosing paradigms, inclusion criteria, and experimental techniques contribute to inconsistent findings across studies. The neuropsychological domains assessed also differ widely—ranging from reaction time and processing speed to subjective cognitive symptoms—often without uniform definitions or the use of standardized testing frameworks [6,10]. Additional variability arises from differences in neuroimaging methodologies, complicating comparisons of functional and structural correlates across cohorts [19,20,21,22]. An added layer of complexity stems from inconsistencies in injection patterns, treatment intervals, and the potential “wear-off phenomenon,” which may influence cognitive symptoms toward the end of each treatment cycle. However, this aspect remains insufficiently explored in cognitive research [32]. Collectively, this methodological diversity hinders the development of a cohesive mechanistic model capable of explaining the cognitive effects of BoNT-A.

## 4. Summary and Implications for Future Research

Taken collectively, these limitations underscore the need for rigorously designed clinical studies explicitly focused on cognition. Future investigations should employ standardized cognitive test batteries, adequately powered sample sizes, carefully controlled assessment timing relative to migraine activity, and multimodal mechanistic approaches to clarify the pathways underlying cognitive change. Large-scale longitudinal trials incorporating neuroimaging and biomarker analyses are essential to determine whether cognitive improvements reflect direct neuromodulatory effects of BoNT-A, secondary benefits resulting from reduced disease burden, or interactions with affective and behavioral factors. As cognitive dysfunction increasingly emerges as a major contributor to CM-related disability, addressing these limitations is critical for advancing both clinical understanding and therapeutic management.

## 5. Conclusions

Current evidence indicates that cognitive dysfunction represents a substantial and clinically meaningful component of chronic migraine, with impairments in attention, processing speed, working memory, and executive functions contributing significantly to overall disability. Emerging data suggest that BoNT-A—already well established as an effective preventive therapy for reducing headache frequency and disease burden—may also provide cognitive benefits through both direct neuromodulatory effects and indirect improvements in affective states and interictal cognitive burden [5,6,8,9,11,17,18]. Recent large-scale observational studies and updated reviews further support the long-term stability of BoNT-A’s therapeutic profile and highlight new research directions, including personalized treatment strategies and combination approaches (e.g., BoNT-A with anti-CGRP monoclonal antibodies), which may also influence cognitive trajectories in patients with refractory disease [33,34,35,36]. However, interpretation of these findings remains limited by methodological heterogeneity, small sample sizes, variability in cognitive assessment protocols, and inadequate control of confounding factors such as anxiety, depression, sleep disturbances, medication overuse, and fluctuations in cognitive functioning across different phases of the migraine cycle [6,7,9]. Moreover, while mechanistic studies provide plausible pathways linking BoNT-A to enhanced cognitive efficiency—such as modulation of cortical excitability, restoration of inhibitory tone, and attenuation of central sensitization—these mechanisms remain largely inferential in the absence of integrated, multimodal research designs [17,18]. Taken together, these limitations underscore the urgent need for rigorously designed, adequately powered studies specifically focused on cognitive outcomes. Future research should incorporate longitudinal neuropsychological testing, standardized assessment timing relative to migraine cycles, and combined neuroimaging and biomarker approaches to determine whether BoNT-A exerts direct cognitive effects, enhances cognition indirectly through improvements in affective or sensory processing, or operates through a combination of these mechanisms. As cognitive dysfunction increasingly emerges as a central driver of disability in chronic migraine, expanding the evidence base in this domain will be essential for informing comprehensive, mechanism-based treatment strategies and optimizing patient-centered outcomes.

## Figures and Tables

**Table 1:** Summary of key cognitive studies.

Study (Year)	Design	Sample (CM Patients)	Cognitive/Relevant Outcomes	Timing	Main Findings
Ho et al., 2020 [8]	Prospective real-world study	60	Reaction time, processing speed, working memory; anxiety, headache	6–12 weeks post-BoNT-A; interictal	Cognitive improvements not correlated with headache reduction
Sánchez-Huertas et al., 2025 [11]	Prospective observational	150	Interictal burden: cognitive slowing, fatigue, photophobia, mood	Across cycles; interictal	Reduced interictal symptoms interfering with cognition
Ilgaz Aydinlar et al., 2024 [7]	Prospective cohort	72	Anxiety, depression, sleep, disability	Baseline vs. follow-up; interictal	Improvements likely enhance cognition indirectly
Santoro et al., 2025 [5]	Long-term real-world	579	Disability, headache days, medication overuse	1–11 years	Stable improvements may support cognitive recovery
Prudenzano, 2024 [12]	Narrative mechanistic review	Not applicable	Mechanistic pathways	—	Biological plausibility for cognitive effects
Bruloy et al., 2019 [13]	Meta-analysis of RCTs	CM across trials	Headache days, disability	Across RCTs	Indirect cognitive relevance via disability reduction

Abbreviations: CM: Chronic Migraine; BoNT-A: OnabotulinumtoxinA; RCTs: Randomized Controlled Trials. Notes: Only Ho et al. (2020) [8] included direct, objective neuropsychological testing. All other cognitive-related findings are indirect, reflecting interictal symptoms, disability, or affective improvements. Evidence suggesting independence from headache reduction is preliminary and based primarily on a single small cohort. Large-scale, standardized cognitive trials in CM with BoNT-A are absent, limiting generalizability.

## Data Availability

No new data were created or analyzed in this study.
